# Protective effects of Guanxin Shutong capsule drug-containing serum on tumor necrosis factor-α-induced endothelial dysfunction through nicotinamide adenine dinucleotide phosphate oxidase and the nitric oxide pathway

**DOI:** 10.3892/etm.2014.1795

**Published:** 2014-06-20

**Authors:** YANJUN CAO, FENG LIU, ZHUANGZHUANG HUANG, YANMIN ZHANG

**Affiliations:** 1 School of Medicine, Xi’an Jiaotong University, Xi’an, Shaanxi 710061, P.R. China; 2 Buchang Pharmaceuticals, Xi’an, Shaanxi 710075, P.R. China

**Keywords:** Guanxin Shutong capsule, tumor necrosis factor-α, nitric oxide, endothelial nitric oxide synthase, nicotinamide adenine dinucleotide phosphate oxidase

## Abstract

The Chinese medicinal formula Guanxin Shutong capsule (GXSTC) has been used for almost 10 years as a clinical treatment for chest pain, depression, palpitation and cardiovascular diseases. The aim of this study was to investigate the effects of GXSTC drug-containing serum on tumor necrosis factor-α (TNF-α)-stimulated endothelial cells. Cell viability was measured by MTT assay, and nitric oxide (NO) levels and NO synthase (NOS) activity were measured as standards of endothelial dysfunction. Malondialdehyde (MDA) levels and superoxide dismutase (SOD) activity were evaluated using commercial kits. In addition, the protein expression of endothelial NOS (eNOS), AKT and nicotinamide adenine dinucleotide phosphate (NADPH) oxidase subunits was examined to evaluate the effect of GXSTC drug-containing serum on ECV304 cells. GXSTC significantly reversed the decrease in NO production induced by TNF-α (5 ng/ml) in ECV304 cells. The expression of NADPH oxidase subunits was increased by TNF-α treatment, but markedly inhibited by treatment with GXSTC in TNF-α-stimulated cells. In summary, GXSTC increased the production of NO in ECV304 cells and exerted a protective effect on ECV304 cells stimulated with TNF-α by upregulating the mRNA and protein expression of eNOS. This was accompanied by increased SOD activity and reduced MDA levels. These results suggested that GXSTC protects the endothelium via the NO pathway and exhibits antioxidant effects.

## Introduction

Cardiovascular disease (CVD) constitutes the leading cause of mortality worldwide. The major modifiable risk factors for ischemic heart disease principally include high blood pressure, high total cholesterol and low-density lipoprotein cholesterol, low high-density lipoprotein cholesterol, tobacco use, physical inactivity, poor nutrition and obesity. All of these established risk factors are known to cause endothelial activation and dysfunction (1,2). The prevention and control of these risk factors are associated with preserved endothelial function and reduced risk of ischemic heart disease.

The normal endothelium exhibits anticoagulant and anti-inflammatory properties, and promotes vasodilatation by the production of nitric oxide (NO), prostacyclin and other vasodilators (3). In various diseases the endothelium can become dysfunctional and promote thrombosis and inflammation, and lose its vasodilatory effects. Endothelial cells (ECs) and the factors released by them play a crucial role in maintaining the physiological functions of the cardiovascular system and are also involved in the development of a variety of human diseases. Oxidative stress is a molecular dysregulation associated with reactive oxygen species (ROS) metabolism, and is a crucial factor in the pathogenesis of endothelial dysfunction, vascular inflammation and atherosclerosis (3). Variations of the phagocytic nicotinamide adenine dinucleotide phosphate (NADPH) oxidases have been identified in all vascular cells, including ECs, vascular smooth muscle cells and fibroblasts (4). The activity and expression of NADPH oxidase can be regulated by cytokines, such as tumor necrosis factor-α (TNF-α), transforming growth factor-β and platelet-derived growth factor. NADPH oxidase activity has been confirmed to be inversely correlated with endothelial function in humans. TNF-α has been shown to stimulate the upregulation of NO synthase (NOS) activity and NO production in ECV304 cells, which may be accompanied by a burst in production of intracellular ROS, including superoxide anion (O_2_^−^) and hydrogen peroxide (5).

ECV304 cells are an abundant and easily accessible EC type. ECV304 cells and human coronary vascular ECs have been shown to exhibit similar sensitivities to the harmful effects of inflammatory cytokines (6), including TNF-α, and oxidative damage. Guanxin Shutong capsule (GXSTC) is a Chinese medicinal formula that is widely used clinically for the treatment of numerous symptoms, including palpitation, restlessness, dyspnea, chest pain, dizziness and fatigue. In addition, GXSTC is used to promote blood circulation, remove blood stasis and prevent CVDs (7,8). Our previous study suggested that oral GXSTC could protect the heart against oxidative stress and apoptosis in rats with myocardial infarction (9). However, the effects of GXSTC on ECs are not well known. The present study was therefore designed to evaluate whether or not GXSTC could protect against TNF-α-induced endothelial dysfunction.

## Materials and methods

### Cell culture

ECV304 cells (Type Culture Collection of the Chinese Academy of Sciences, Shanghai, China) were grown in Dulbecco’s modified Eagle’s medium (DMEM) containing 10% bovine serum, antibiotics (100 IU/ml penicillin and 100 mg/ml streptomycin), at 37°C in a 5% CO_2_ atmosphere.

### Drug and reagents

GXSTC consists of five traditional Chinese drugs: 57.1% Fructus Choerospondiatis (Polyphagidae), 28.6% *Salvia miltiorrhiza* (Labiatae), 7.1% *Syzygium aromaticum* (Labiatae), 3.6% borneol (Leguminosae) and 3.6% tabasheer. GXSTC (batch no. 20120125) was provided by Buchang Pharmaceuticals (Xi’an, China). DMEM and fetal bovine serum were obtained from Gibco-BRL (Grand Island, NY, USA). The recombinant human TNF-α was obtained from Promega Corp. (Madison, WI, USA). The anti-endothelial NOS (eNOS) and anti-AKT monoclonal antibodies were purchased from Cell Signaling Technology, Inc. (Boston, MA, USA). Antibodies for p47phox and GAPDH were purchased from Santa Cruz Biotechnology, Inc. (Santa Cruz, CA, USA) and those for gp91phox and NAPDH oxidase 4 (Nox4) were purchased from Epitomics (Burlingame, CA, USA). Enhanced chemiluminescence reagent was obtained from Pierce Biotechnology, Inc. (Rockford, IL, USA). All primers used were provided by Takara Bio, Inc. (Shiga, Japan), and the NO, NOS, superoxide dismutase (SOD), malondialdehyde (MDA) and lactate dehydrogenase (LDH) assay kits were purchased from the Nanjing Jiancheng Bioengineering Institute (Nanjing, China).

### Preparation of serum containing the tested drugs

Subsequent to obtaining approval from the Ethics Committee of Xi’an Jiaotong University (Xi’an, China), male Sprague Dawley rats weighing 200–250 g were provided by the Experimental Animal Center of Xi’an Jiaotong University School of Medicine and housed in a room with a temperature of 21–25°C, a relative humidity of 50–60% and a 12-h light/dark cycle. Following the removal of the capsules, the GXSTC powder was dissolved in aseptic 0.5% sodium carboxymethylcellulose. The study was conducted in accordance with the Guidelines for the Care and Use of Laboratory Animals of Xi’an Jiaotong University. Each group contained 10 rats and they were used for the preparation of the serum containing the tested drugs. Rats in the normal and low-, medium- and high-dose GXSTC serum groups were intragastrically administered saline or 5, 10 or 20 g/kg GXSTC, respectively, for 14 days. Blood was aseptically obtained from the abdominal aorta of the rats 2 h after the final administration and the serum was then acquired by centrifugation of the blood at 720 × g for 20 min. Following filtration twice with a 0.22-μm cellulose acetate membrane, the serum was bottled, calefied in 56°C water for 30 min and stored at −20°C for use (10). ECV304 cells were subsequently divided into five groups, as follows: GXSTC-L (treatment with 5 ng/ml TNF-α and GXSTC at 5 g/kg), GXSTC-M (treatment with 5 ng/ml TNF-α and GXSTC at 10 g/kg), GXSTC-H (treatment with 5 ng/ml TNF-α and GXSTC at 20 g/kg), TNF-α (treatment with 5 ng/ml TNF-α and vehicle) and control (treatment with vehicle but without TNF-α).

### Measurement of NO levels and NOS activity

Subsequent to the ECV304 cells reaching confluence in a 96-well plate, DMEM containing various concentrations of TNF-α (2.5, 5 and 10 ng/ml) was added. After 6 h of culture, the medium from each sample was collected, and the levels of NO released by the ECV304 cells and the NOS activity were calculated using the NO and NOS assay kits according to the manufacturer’s instructions (Nanjing Jiancheng Bioengineering Institute).

In order to determine the effects of GXSTC on NO levels and NOS activity in TNF-α-stimulated ECV304 cells, the cells were again cultured in a 96-well plate and were then treated with the different concentrations of GXSTC drug-containing serum (low-, medium- and high-dose GXSTC) for 24 h followed by treatment with TNF-α (5 ng/ml) for 6 h. In all the groups, including the control group, the same volume of vehicle serum in the DMEM was added. The medium from each sample was collected after 6 h and the levels of NO released by the ECV304 cells and the NOS activity were calculated using the assay kits.

### MTT assay for cell viability

Cell survival was quantified by the colorimetric MTT assay (Sigma, St. Louis, MO, USA), which measures mitochondrial activity in viable cells. Briefly, cells in the exponential growth period were harvested and plated in 96-well plates at a concentration of 1×10^4^ cells/well, and incubated at 37°C for 24 h. The cells were treated with the different concentrations of GXSTC drug-containing serum (low-, medium- and high-dose GXSTC) for 24 h, and subsequently treated with TNF-α (5 ng/ml) for 6 h. MTT (5 mg/ml, 20 μl) was then added to each well and the cells were incubated at 37°C for 4 h. Subsequent to the supernatant being discarded, 150 μl dimethylsulfoxide was added to each well, and the absorbance at 490 nm was determined by use of a microplate reader (Bio-Rad, Hercules, CA, USA).

### Assay for LDH release

Cytotoxicity was quantified by measuring LDH release in the medium during the exposure to different reagents (11). Subsequent to ECV304 cells reaching confluence in a 96-well plate, the cells were treated with the different concentrations of GXSTC drug-containing serum (low-, medium- and high-dose GXSTC) for 24 h and then treated with TNF-α (5 ng/ml) for 6 h.

### Determination of MDA levels and SOD activity

Subsequent to ECV304 cells reaching confluence in a 96-well plate, the cells were treated with the different concentrations of GXSTC drug-containing serum (low-, medium- and high-dose GXSTC) for 24 h and then treated with TNF-α for 6 h. The total SOD activity and MDA levels in the cell lysates were assayed using reagent kits in accordance with the manufacturer’s instructions (Nanjing Jiancheng Bioengineering Institute) (12).

### RNA preparation and semi-quantitative reverse transcription polymerase chain reaction (RT-PCR)

To determine mRNA levels, confluent ECV304 cells grown in six-well plates were treated as previously described (12). Total RNA was extracted using TRIzol^®^ (Invitrogen Life Technologies, Carlsbad, CA, USA) according to the manufacturer’s instructions. cDNA samples were generated and amplified using a Takara RNA PCR kit (avian myeloblastosis virus) Ver. 3.0 (Takara Bio, Inc.) following the manufacturer’s instructions. Thirty-five cycles of PCR amplification were performed for eNOS, inducible NOS (iNOS), neuronal NOS (nNOS) and GAPDH (95°C for 35 sec, 62°C for 90 sec and 72°C for 90 sec) using specific primers (Table I). The PCR products were visualized on 1.5% agarose gels stained with GoldView™ (Science and Technology Ltd, Beijing, China) staining. GAPDH served as an internal control.

### Western blotting

Confluent ECV304 cells in six-well plates were treated with GXSTC and then with TNF-α in accordance with the aforementioned methods. The protein expression of AKT, eNOS, p47phox, gp91phox, Nox4 and GAPDH was measured by western blot analysis as previously described (13).

### Statistical analysis

Values are presented as the mean ± standard error of the mean. One-way analysis of variance followed by a Tukey’s multiple comparison test were used to test the significance between three or more groups. P<0.05 was considered to indicate a statistically significant difference.

## Results

### Effects of GXSTC on NO production by ECV304 cells stimulated with TNF-α

**Figure 1:**
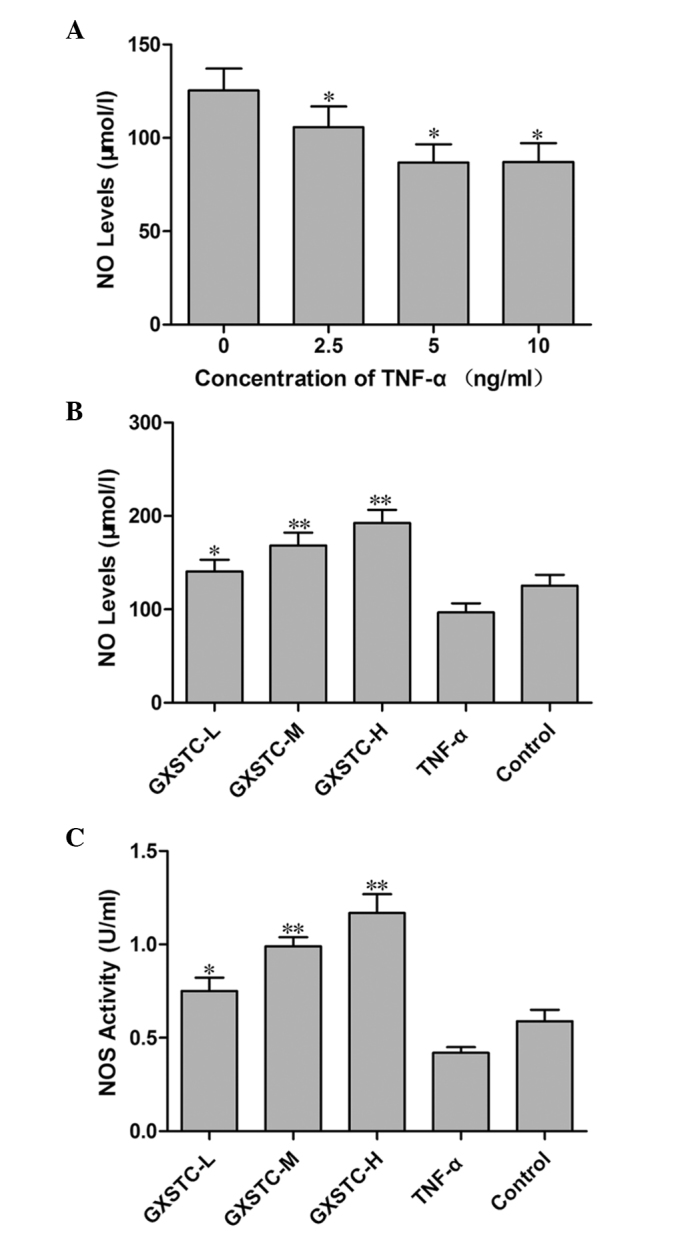
Levels of NO and activity of NOS in the endothelial cell culture medium. (A) Effects of TNF-α on NO levels in ECV304 cells. (B and C) Effects of GXSTC on (B) NO levels and (C) NOS activity in ECV304 cells stimulated with TNF-α. Dulbecco′s modified Eagle′s medium containing the indicated concentration of TNF-α alone or in the presence of the GXSTC was added to 90% confluent ECV304 cells in a 12-well plate. The concentration of NO released into the medium is presented as the mean ± standard error of the mean. ^*^P<0.05 and ^**^P<0.01 vs. the 0 ng/ml group. NO, nitric oxide; NOS, nitric oxide synthase; TNF-α, tumor necrosis factor-α; GXSTC, Guanxin Shutong capsule; GXSTC-L, low-dose GXSTC; GXSTC-M, medium-dose GXSTC; GXSTC-H, high-dose GXSTC.

TNF-α treatment significantly reduced the NO production by ECV304 cells, indicating that endothelial dysfunction had occurred. The concentration of 5 ng/ml TNF-α was selected as an *in vitro* endothelial dysfunction model (Fig. 1A), and GXSTC increased the level of NO in a dose-dependent manner in TNF-α-stimulated ECV304 cells (Fig. 1B). Compared with cells in the TNF-α group, the activity of NOS was significantly increased in the cells following treatment with GXSTC (P<0.05, Fig. 1C). NO is generated by three NOSs, and the mRNA expression of these three NOSs was measured in each experiment using RT-PCR. The expression of eNOS was decreased in the TNF-α-treated group but increased following incubation with GXSTC. The expression of iNOS was increased in the TNF-α-treated group but decreased after incubation with GXSTC, whereas nNOS were not detected by amplification

### Effects of GXSTC on cell viability of ECV304 cells stimulated with TNF-α

**Figure 3:**
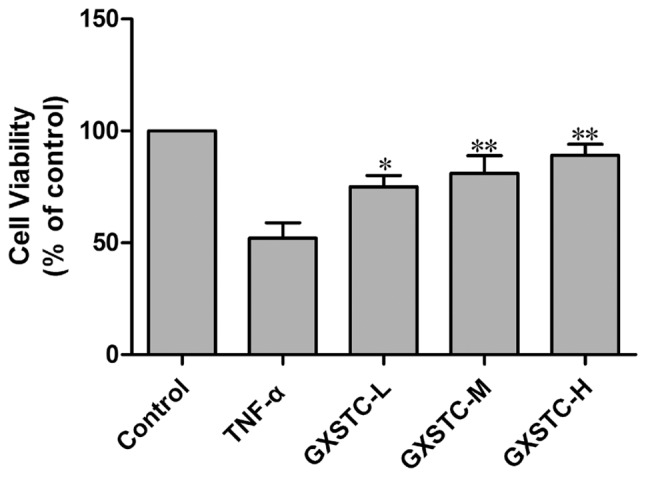
Cell viability. Cultured ECV304 cells were either left untreated (control) or treated with TNF-α (5 ng/ml) alone or in the presence of the GXSTC for 24 h. The results are presented as the mean ± standard error of the mean of five individual experiments. ^*^P<0.05 and ^**^P<0.01 vs. the TNF-α group. TNF-α, tumor necrosis factor-α; GXSTC, Guanxin Shutong capsule; GXSTC-L, low-dose GXSTC; GXSTC-M, medium-dose GXSTC; GXSTC-H, high-dose GXSTC.

The exposure of cells to TNF-α for 6 h induced cell death in more than one-third of the cells as compared with the control group, as measured by the MTT assay (Fig. 3). GXSTC significantly attenuated TNF-α-induced cell death.

### Effects of GXSTC on LDH release by ECV304 cells stimulated with TNF-α

**Figure 4:**
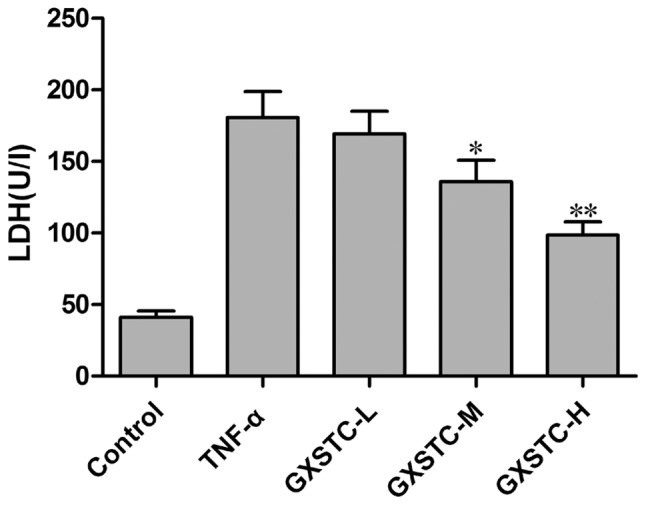
LDH activity, reflecting cellular damage. Cultured ECV304 cells were either left untreated (control) or treated with TNF-α (5 ng/ml) alone or in the presence of GXSTC for 24 h. The results are presented as the mean ± standard error of the mean of five individual experiments. ^*^P<0.05 and ^**^P<0.01 vs. the control group. LDH, lactate dehydrogenase; TNF-α, tumor necrosis factor-α; GXSTC, Guanxin Shutong capsule; GXSTC-L, low-dose GXSTC; GXSTC-M, medium-dose GXSTC; GXSTC-H, high-dose GXSTC.

As shown in Fig. 4, LDH release was minimal in the control group. The stimulation of cells with TNF-α resulted in a marked increase in LDH release. GXSTC significantly attenuated the TNF-α-induced increase in LDH release.

### Effects of GXSTC on MDA levels and SOD activity in ECV304 cells stimulated with TNF-α

**Figure 5:**
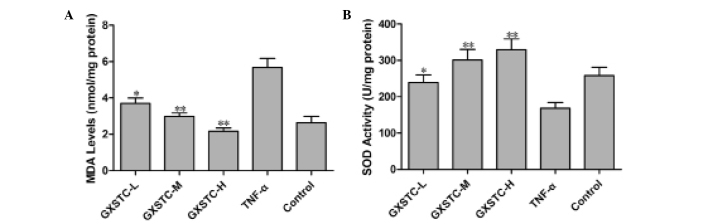
Effects of GXSTC on (A) production of the lipid peroxidation product MDA and (B) SOD activity in endothelial cells stimulated with TNF-α. Cultured ECV304 cells were either left untreated (control) or treated with TNF-α (5 ng/ml) alone or in the presence of GXSTC for 24 h. The results are presented as the mean ± standard error of the mean of five individual experiments. ^*^P<0.05 and ^**^P<0.01 vs. the TNF-α group. GXSTC, Guanxin Shutong capsule; TNF-α, tumor necrosis factor-α; MDA, malondialdehyde; SOD, superoxide dismutase; GXSTC-L, low-dose GXSTC; GXSTC-M, medium-dose GXSTC; GXSTC-H, high-dose GXSTC.

GXSTC treatment resulted in significant decreases in MDA content (P<0.05, Fig. 5A) and increases in SOD activity (P<0.05, Fig. 5B) in ECV304 cells exposed to TNF-α as compared with cells exposed to TNF-α alone.

### Effects of GXSTC on protein expression of eNOS in ECV304 cells stimulated with TNF-α

**Figure 6:**
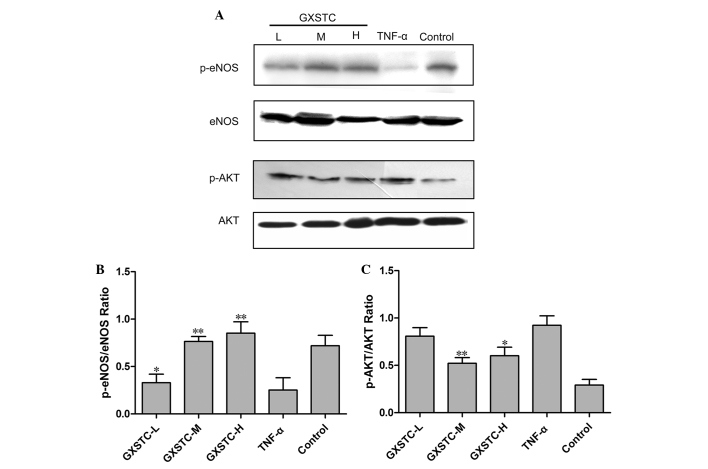
Effects of GXSTC on eNOS and AKT protein expression in TNF-α-stimulated endothelial cells. ECV304 cells were grown to confluence in six-well plates and pre-treated with GXSTC for 24 h. A total of 5 ng/ml TNF-α was then added for 6 h. (A) Bands corresponding to eNOS, p-eNOS, AKT and p-AKT. (B and C) The results for (B) p-eNOS and (C) p-AKT were quantified by densitometric analysis of the bands and then normalized to eNOS and AKT, respectively, in ECV304 cells. Data are presented as the mean ± standard error of the mean of three independent experiments duplicated in each run. ^*^P<0.05 and ^**^P<0.01 vs. the TNF-α group. GXSTC, Guanxin Shutong capsule; eNOS, endothelial nitric oxide synthase; TNF-α, tumor necrosis factor-α; p-, phosphorylated-; GXSTC-L, low-dose GXSTC; GXSTC-M, medium-dose GXSTC; GXSTC-H, high-dose GXSTC.

Exposure of cells to TNF-α for 6 h resulted in a significant decrease in eNOS protein expression (P<0.05, Fig. 6); GXSTC restored eNOS protein expression (Fig. 6). Treatment of ECV304 cells with TNF-α for 6 h induced the phosphorylation of AKT. However, when cells were preincubated with GXSTC for 6 h, the phosphorylation of AKT decreased in a dose-dependent manner (Fig. 6).

### Effects of GXSTC on NADPH oxidase expression in ECV304 cells stimulated with TNF-α

**Figure 7:**
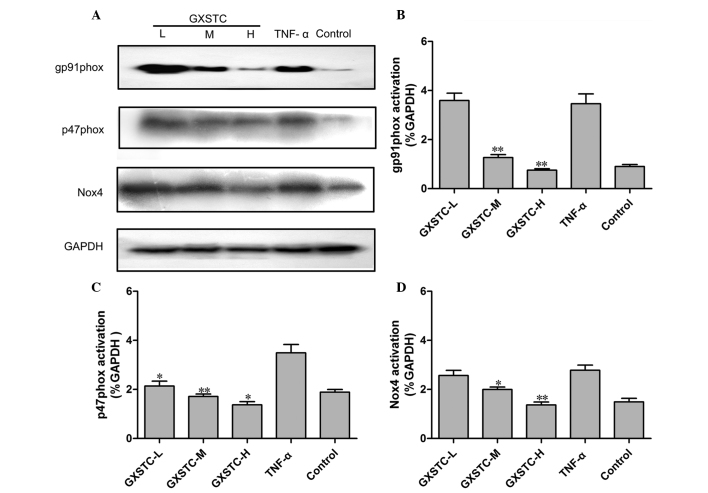
Effects of GXSTC on NADPH oxidase protein expression in TNF-α-stimulated endothelial cells. ECV304 cells were grown to confluence in six-well plates and pre-treated with GXSTC for 24 h. A total of 5 ng/ml TNF-α was then added for 6 h. (A) Bands corresponding to gp91phox, p47phox, Nox4 and GAPDH. (B–D) The results for (B) gp91phox, (C) p47phox and (D) Nox4 were quantified by densitometric analysis of the bands and then normalized to GAPDH in ECV304 cells. Data are presented as the mean ± standard error of the mean (n=3). ^*^P<0.05 and ^**^P<0.01 vs. the TNF-α group. GXSTC, Guanxin Shutong capsule; NAPDH, nicotinamide adenine dinucleotide phosphate; TNF-α, tumor necrosis factor-α; Nox4, NAPDH oxidase 4; GXSTC-L, low-dose GXSTC; GXSTC-M, medium-dose GXSTC; GXSTC-H, high-dose GXSTC.

Oxidative stress and increased O_2_^−^ production by NADPH oxidases are critical in the development of CVDs. The expression of p47phox, gp91phox and Nox4 protein was increased in TNF-α-stimulated ECV304 cells. Administration of GXSTC significantly decreased p47phox, Nox-4, and gp91phox protein expression compared with TNF-α treatment alone (Fig. 7).

## Discussion

In the present study, TNF-α stimulation resulted in reduced cell viability and increased the release of LDH and the lipid peroxidation product MDA; this was associated with increased production of NO and NADPH oxidase, suggesting that underproduction of NO and downregulation of p-eNOS may have partially mediated the cell death through enhancing oxidative stress and consequently increasing the cellular lipid peroxidation. TNF-α stimulation resulted in cellular injury and reduced NO production in ECV304 cells, and co-culture of ECV304 cells with GXSTC conferred protection against cellular injury and significantly increased NO production. The GXSTC-induced enhancement of NO production appeared to be attributed to the restoration of eNOS production. GXSTC enhanced cell viability and reduced the cellular LDH release induced by TNF-α. This phenomenon may suggest that underproduction of NO by ECs in response to TNF-α stimulation had the initial function of cell protection rather than injury. However, under circumstances where the ensuing burst in ROS production induced by TNF-α is not counteracted by sufficient antioxidant defense, the outcome may be endothelial injury. In the present study, GXSTC significantly attenuated the TNF-α-induced reduction in the activity of SOD, a major endogenous antioxidant, and attenuated the TNF-α-induced increase in the lipid peroxidation product MDA. These findings may represent major mechanisms by which GXSTC attenuates TNF-α-induced endothelial injury.

The modulation of vascular tone and structure is reliant on the multifunctional vascular endothelium (2). Cellular adhesion, thromboresistance, smooth muscle cell proliferation and vessel wall inflammation are regulated by a variety of factors produced by ECs (1). Thus, since the endothelium plays a critical role in maintaining the homeostasis of the body, endothelial dysfunction is associated with a number of pathophysiological conditions, including atherosclerosis, hypertension, diabetes and certain CVDs. There are three distinct isoforms of NOS, which differ both in structure and in function. eNOS and nNOS are constitutively expressed and are referred to as Ca^2+^-dependent enzymes. iNOS is only expressed at high levels following induction by cytokines or other inflammatory agents, and its activity is independent of an increase in intracellular Ca^2+^ (14). The main source of endothelial NO is eNOS expressed by ECs. In particular, NO plays a key role in protecting the endothelium against the initiation and progression of CVD via its dilatory effect (2), and may also be involved in cardioprotection (15). Since it protects against CVDs, NO production by ECV304 cells was selected as an indicator of endothelial dysfunction in the present study. The nNOS isoform could not be detected by amplification. Therefore, the effects of treatment with GXSTC drug-containing serum on NO production in TNF-α-treated ECV304 cells were determined by measuring NO generation, NOS activity and eNOS expression. TNF-α decreased the secretion of NO into the medium, but GXSTC increased eNOS expression and enhanced NO production and NOS activity. These results suggest that TNF-α-stimulated ECV304 cells represent an endothelial dysfunction model, and that GXSTC can improve the endothelial dysfunction by inducing eNOS expression and increasing NO production and NOS activity.

Endothelial NO is produced by eNOS in response to physiological stimuli. In the absence of its substrate (L-arginine), the eNOS generates O_2_^−^ instead of NO. NO exerts a number of beneficial antiatherogenic effects and reacts with a variety of other targets, acting to modulate ion channel function, intracellular signal transduction and gene expression (7,16). Therefore, the loss of NO bioavailability is a key feature of endothelial dysfunction, and can occur due to multiple pathways altering NO synthesis or biodegradation. The excessive production of ROS appears to be a major mechanism underlying reduced vascular NO levels. There are several mechanisms through which NO bioavailability can be modified by ROS (2). Firstly, O_2_^−^ rapidly reacts with NO, leading to the production of the strong oxidant peroxynitrite. Treatment with SOD or SOD mimetics reduces vascular O_2_^−^ levels and restores endothelial function (1). NADPH oxidases, lipoxygenase, xanthine oxidase, mitochondrial oxidases and NOSs are potential sources of vascular O_2_^−^ production (4,17,18). The NADPH oxidase has been shown to be the principal source of O_2_^−^ production in certain animal models of vascular disease. The enzyme was originally characterized in neutrophils, and has been shown to be present in vascular smooth muscle cells and ECs (19). Furthermore, the nitration and nitrosylation of the NADPH oxidase may lead to the inhibition of the enzyme. In the present study, NADPH oxidase and AKT expression was inhibited subsequent to NOS activity being enhanced by GXSTC. This association remained even following correction for other major risk factors for atherosclerosis, including diabetes, hypercholesterolemia and coronary heart disease.

In conclusion, GXSTC inhibited the expression of p47phox, gp91phox and Nox4, which are NADPH oxidase subunits and major risk factors for CVDs. Furthermore, GXSTC was shown to reduce SOD activity in TNF-α-stimulated ECV304 cells. eNOS mRNA and protein levels in ECV304 cells were increased by GXSTC. Previous studies have indicated that GXSTC exerts an anti-ischemic effect during the early and late stages of myocardial infarction (20,21). This study supports the hypothesis that GXSTC protects against endothelial injury via the NO pathway and its antioxidant effects. GXSTC may have an important role in preventing atherosclerosis or myocardial infarction initiated by endothelial injury.

## Figures and Tables

**Figure 2:**
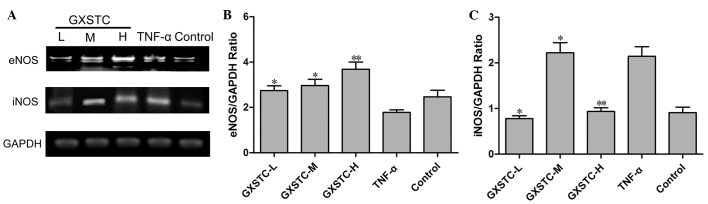
Effects of GXSTC on the mRNA expression of eNOS, iNOS and nNOS by ECV304 cells stimulated with TNF-α. ECV304 cells were grown to confluence in six-well plates and pre-treated with GXSTC for 24 h. A total of 5 ng/ml TNF-α was then added for 6 h. (A) Bands corresponding to eNOS, iNOS and GAPDH. (B and C) The results for (B) eNOS and (C) iNOS were quantified by densitometric analysis of the bands and then normalized to GAPDH in ECV304 cells. Data are presented as the mean ± standard error of the mean (n=3). ^*^P<0.05 and ^**^P<0.01 vs. the TNF-α group). GXSTC, Guanxin Shutong capsule; GXSTC-L, low-dose GXSTC; GXSTC-M, medium-dose GXSTC; GXSTC-H, high-dose GXSTC; eNOS, endothelial nitric oxide synthase; iNOS, inducible NOS; nNOS, neuronal NOS; TNF-α, tumor necrosis factor-α.

**Table I:** Primers used for eNOS, iNOS, nNOS and GAPDH.

mRNA	Primer	Sequence (5′-3′)
eNOS	Sense	CACCGCTACAACATCCTG
	Antisense	GCCTTCTGCTCATTCTCC
iNOS	Sense	GCTACCAGATGCCAGATG
	Antisense	CTCAAGCACAAGGTCAGG
nNOS	Sense	GTGGAGGTGCTGGAGGAG
	Antisense	GTGCGGTAGGAAACGATGG
GAPDH	Sense	CACCCACTCCTCCACCTTTG
	Antisense	CCACCACCCTGTTGCTGTAG

eNOS, endothelial nitric oxide synthase; iNOS, inducible NOS; nNOS, neuronal NOS.
